# The Spatial Distribution of Hepatitis C Virus Infections and Associated Determinants—An Application of a Geographically Weighted Poisson Regression for Evidence-Based Screening Interventions in Hotspots

**DOI:** 10.1371/journal.pone.0135656

**Published:** 2015-09-09

**Authors:** Boris Kauhl, Jeanne Heil, Christian J. P. A. Hoebe, Jürgen Schweikart, Thomas Krafft, Nicole H. T. M. Dukers-Muijrers

**Affiliations:** 1 Department of Health, Ethics and Society, School of Public Health and Primary Care (CAPHRI), Faculty of Health, Medicine and Life Sciences, Maastricht University, Maastricht, the Netherlands; 2 Department of Sexual Health, Infectious Diseases and Environmental Health, South Limburg Public Health Service (GGD Zuid Limburg), Geleen, The Netherlands; 3 Department of Medical Microbiology, School of Public Health and Primary Care (CAPHRI), Maastricht University Medical Center (MUMC+), Maastricht, The Netherlands; 4 Beuth University of Applied Sciences, Department III, Civil Engineering and Geoinformatics, Berlin, Germany; University of North Carolina School of Medicine, UNITED STATES

## Abstract

**Background:**

Hepatitis C Virus (HCV) infections are a major cause for liver diseases. A large proportion of these infections remain hidden to care due to its mostly asymptomatic nature. Population-based screening and screening targeted on behavioural risk groups had not proven to be effective in revealing these hidden infections. Therefore, more practically applicable approaches to target screenings are necessary. Geographic Information Systems (GIS) and spatial epidemiological methods may provide a more feasible basis for screening interventions through the identification of hotspots as well as demographic and socio-economic determinants.

**Methods:**

Analysed data included all HCV tests (n = 23,800) performed in the southern area of the Netherlands between 2002–2008. HCV positivity was defined as a positive immunoblot or polymerase chain reaction test. Population data were matched to the geocoded HCV test data. The spatial scan statistic was applied to detect areas with elevated HCV risk. We applied global regression models to determine associations between population-based determinants and HCV risk. Geographically weighted Poisson regression models were then constructed to determine local differences of the association between HCV risk and population-based determinants.

**Results:**

HCV prevalence varied geographically and clustered in urban areas. The main population at risk were middle-aged males, non-western immigrants and divorced persons. Socio-economic determinants consisted of one-person households, persons with low income and mean property value. However, the association between HCV risk and demographic as well as socio-economic determinants displayed strong regional and intra-urban differences.

**Discussion:**

The detection of local hotspots in our study may serve as a basis for prioritization of areas for future targeted interventions. Demographic and socio-economic determinants associated with HCV risk show regional differences underlining that a one-size-fits-all approach even within small geographic areas may not be appropriate. Future screening interventions need to consider the spatially varying association between HCV risk and associated demographic and socio-economic determinants.

## Introduction

Hepatitis C virus (HCV) infections are a major cause of liver diseases and are the leading cause for liver cirrhosis worldwide [1]. The World Health Organization estimates that 123 million people globally are infected with HCV [2]. A major challenge for public health response to HCV is its mostly asymptomatic nature and therefore the limited number of HCV positive individuals aware of their HCV status. Infected, but undiagnosed persons are an important source for further transmission [3]. Several studies estimated the proportion of asymptomatic infections to account for 70% [4,5] to 90% [6] of acute infections, leading to only a small proportion of infected individuals seeking medical attention for symptoms related to HCV infection [7]. It is estimated that less than one-third of HCV infected individuals are aware of their HCV status [8–10]. Many infections are either undetected or are detected at a late stage. Highly effective therapeutic options for HCV are becoming available, [11,12] but logically only to persons, who’s HCV infection is diagnosed.

To provide an opportunity for treatment of HCV positive persons, which are yet undiagnosed and therefore currently hidden to care, preventive screening is necessary.

The HCV prevalence and its associated risk factors varies considerable between countries [13,14]. Past interventions focused on the population in general were not very cost-effective, especially in countries where the overall HCV prevalence is low. In the Netherlands, the HCV prevalence in the Dutch adult population is estimated to be relatively low with 0.2%, although estimates vary between 0.1 and 0.4%, depending largely on the study design and population studied [15,16]. A meta analysis on the effectiveness of screening interventions suggests that for low HCV prevalence populations, pre-screening selection criteria should be used to increase efficiency [17]. The World Health Organisation (WHO) recommends in its new guidelines to offer HCV tests to people with high risk behaviour and to people from high risk populations [18]. These target populations include transmission risk groups such as injecting drug users (IDU) [5,10,11], blood transfusion recipients [3], surgery and dialysis patients [13], professionals in patient care [5], immigrants from endemic countries [13], persons with low socio-economic status [5] and HIV positive men who have sex with men (MSM) [3]. However, screening approaches to target these risk groups have not been shown to be effective in revealing the totality of hidden cases as the identification of people who belong to such risk groups in the first place appeared to be quite challenging. Furthermore, in the Netherlands it had been shown that a substantial part (25%) of all HCV infections is not attributable to any of the aforementioned risk groups and is therefore not included in screening interventions targeted at risk groups [16]. Although the prevalence of HCV in the US is higher with an estimated 2% [19], the Center for Disease Control (CDC) similar to the WHO advises screening of persons in risk groups (IDU, blood transfusion or organ transplant recipients before July 1992, health care personnel with history of exposure and born to an HCV-positive mother) [20]. However, these criteria appeared also in the US difficult to include in practical screening interventions [10]. As a result, future screening interventions need to find characteristics of HCV that are more practically applicable than the risk groups and behavioural factors outlined above.

Other relevant factors than behavioural and demographic risk factors associated with HCV are socio-economic characteristics. As for many infectious diseases, including HCV, lower socio-economic status tends to be associated with higher prevalence [1,13,21,22]. The identification of socio-economic determinants provides a more practically applicable basis for screening interventions [10], as population characteristics are typically available within population data [23]. The application of Geographic Information Systems (GIS) is essential to display the spatial heterogeneity of disease risk and to quantify the impact of socio-economic determinants on the incidence of infectious diseases [22,24].

Exploratory disease mapping and local cluster tests have shown to help identifying areas with statistically significant high risks (often referred to as hotspots or clusters) for prioritizing future interventions for Hepatitis C in the mainland of China [25] as well as many other infectious diseases including HIV [26], *Chlamydia trachomatis* and *Neisseria gonorrhea* [27].

The increasing availability of a wide range of population-based variables allows a detailed analysis of demographic and socio-economic determinants of disease risk using spatial regression models at the ecological level [24,28,29].

With respect to HCV, it has been shown that not only prevalence varies between and within countries, but also the association between risk factors and HCV prevalence [13], highlighting the necessity to account for local variation in spatial regression models for HCV.

In settings where strong local variation of the association between disease risk and possible determinants can be expected, geographically weighted Poisson regression models (GWPR) have proven to be very effective to measure the spatially varying association between possible determinants and disease risk. This in turn, often led to the conclusion that the determinants of a specific disease depend largely where infected populations live, allowing public health preventions to be targeted directly at those population groups, that are most at risk in a specific location [30–32].

The aim of this paper is therefore to (i) determine hotspots for future screening interventions using the spatial scan statistic and (ii) to assess demographic and socio-economic determinants of HCV risk within these hotspots using GWPR to facilitate targeted, evidence-based screening interventions aimed directly at risk-groups.

## Data and Methods

### Ethics Statement

The medical ethics committee of the Maastricht University Medical Centre (Maastricht, the Netherlands) approved the study (11-4-136) and waived the need for consent to be collected from participants. Since retrospective data originated from standard care (in which one can opt-out for the use of their data for scientific research) and were analyzed anonymously, no further informed consent for data analysis was obtained.

### Dependent Variable

The dependent variable consisted of the HCV diagnoses that were made in the southern part of the province Limburg, the Netherlands between January 1st, 2002 and December 31^st,^ 2008, comprising an adult population of 500,955 in 2008 [10,33]. The diagnoses were retrieved from HCV test data that were provided by three hospital laboratories, which perform tests on HCV upon request of nearly all care providers serving the area. In total 23,800 HCV tests were conducted of which 823 unique patients were tested positive. According to screening procedures in the Netherlands, HCV antibodies were detected with an ELISA. Confirmation was performed with an immunoblot and/or polymerase chain reaction (PCR). When an acute infection was suspected or when the patient was HIV positive or on hemodialysis, only PCR was used for screening. In the current study, we defined a positive confirmation test or PCR as a positive case. Of these 823 unique positive individuals, 781 had valid postal codes assigned and were included in the analysis. Next to postal code and HCV test result, the laboratory dataset included sex and age [10].

### Explanatory Variables

We assessed several demographic and socio-economic variables for their association with HCV risk. The data for these variables were downloaded from the Central Bureau for Statistics Netherlands. In this study, we used data and map sources from the Statline database 2009 [33] (Table 1). The data were available on neighbourhood level and had to be matched to the four-digits postal codes of the HCV data. A neighbourhood is a part of a municipality with a homogenous socio-economic structure [33,34]. Due to privacy restrictions, socio-economic data on neighbourhood level is only available for neighbourhoods with more than 50 persons, 200 persons, 10 households and 70 households, depending on the respective variable [33]. We therefore aggregated to the four-digits postal codes based on those neighbourhoods, for which socio-economic data was made available.

**Table 1:** Explanatory variables.

Variable	Average	Min	Max
**Married (%)**	44.7	1.0	62.0
**Unmarried (%)**	40.9	96.0	15.0
**Divorced (%)**	3.8	0.0	10.6
**Widowed (%)**	6.8	0.0	48.0
**Non-western immigrants (%)**	4.8	0.0	14.2
**One-person households (%)**	36.4	7.0	71.8
**Households w/o children (%)**	32.1	20.7	57.0
**Social welfare recipients (%)**	1.2	0.5	14.6
**Average income (in 1000 Euro)**	20.5	14.2	29.1
**Persons with low income (%)**	2.1	0.5	15.5
**HH with low purch. power (%)**	8.9	0.0	20.0
**Households with low income (%)**	45.9	17.0	67.0
**HH below social minimum (%)**	9.3	0.0	22.0
**Mean prop. value (in 1000 Euro)**	213.6	116.7	433
**Males aged 36–45 (%)**	6.4	0.0	12.2
**Males aged 46–55 (%)**	8.2	0.0	11.2

Demographic variables included stratified population data for 2012 on four-digits postal code level [10] [16]. The population data was extracted from customised data by Statistics Netherlands (Extraction date: 20/02/2013).

Socio-economic variables included marital status (proportion of residents that were married, unmarried, divorced, or widowed)[35], proportion of non-western immigrants [16], proportion of one-person households, proportion of households without children, average income [10,36], proportion of persons having low income [36] (defined as an income below 19,200 Euro per year [33]), households having low purchasing power (defined as households having less than 9,250 Euro available per year [33]), households having low income [36] (defined as households with an annual income below 25,100 Euro [33]), households below social minimum and mean property value as indicator for potential area deprivation [10,33].

### Exploratory Disease Mapping

We calculated the prevalence rate of HCV and the relative risk (RR) for the adult population aged between 16 and 65.

The RR estimates provide useful information how common HCV infection in a specific location is as compared to the global baseline [37]. We additionally applied spatial empirical Bayes smoothing since the population at risk displayed strong regional variation. This leads to a large variance of the prevalence rate and the relative risk especially in areas where the underlying population is small [38]. Due to strong regional variation in the HCV prevalence, we applied a local smoothing approach. The prevalence rates and the RR were therefore smoothed towards a local mean where the neighbours were defined as areas sharing a common edge and a common boundary [39]. The calculation of the spatial empirical Bayes smoothing was carried out using OpenGeoDa 1.2.0 [40] and the results were then imported in ESRI ArcGIS 10.1.

### Global Cluster Detection

To test whether there is spatial autocorrelation of the HCV prevalence, we used Moran`s I. Moran`s I is a widely used global cluster test, which determines the degree of clustering or dispersion within a data set. The resulting values may range from 1 (perfect correlation), 0 (complete spatial randomness) to -1 (perfect dispersed) [41]. For the HCV data, a positive spatial autocorrelation means that postal code areas with high HCV prevalence are close to other postal code areas with high HCV prevalence. For this study, we defined adjacency as postal code areas sharing a common edge or corner. The presence of global clustering justified the subsequent local cluster analysis. The computation of Moran`s I was carried out in OpenGeoDa 1.2.0 [40]

### Local Cluster Detection

The spatial scan statistic has been widely applied in several spatial-epidemiological studies to detect local clusters with statistically significant elevated risk of infectious diseases [22,26,42,43]. The spatial scan statistic is a local cluster test, which identifies the location and the statistical significance of local clusters [26]. We applied a Poisson purely spatial model where the number of HCV cases follows an inhomogeneous Poisson process [44]. The input data for this model consisted of the number of positive individuals per postal code, the number of adults aged between 16 and 65 and the centroid coordinates for each area. The spatial scan statistic imposes a circular scanning window, which is flexibly in size and position and gradually moves over all coordinates, evaluating all potential cluster locations and sizes up to either a user-defined maximum radius, a user defined maximum percentage of the population at risk or the default value of up to 50% of the population at risk [45].

In our study, the purpose of the spatial scan statistic was to detect areas with significantly elevated risk of diagnosed HCV, which can serve as a basis for the prioritization of future screening interventions [46,47]. We set the maximum population at risk to not exceed 5% of the adult population. This was done to detect local clusters as precisely as possible since the default settings of 50% of the population at risk are more likely to produce clusters of no practical use [48]. The computation was carried out using the SaTScan software version 9.2 [45].

### Spatial Regression

#### Ordinary Least Squares Regression

To specify a meaningful geographically weighted Poisson regression model, we conducted several steps: First, we performed a natural log-transformation of the dependent variable. We then used a data-mining tool called Exploratory Regression in ESRI ArcGIS 10.1. to determine potential candidate explanatory variables. This tool evaluates all possible variable combinations that form a properly specified ordinary least squares (OLS) regression model. Exploratory regression is comparable to a step-wise regression [31]. However, it evaluates all possible variable combinations based on following criteria: (i) the coefficients are statistically significant, (ii): the explanatory variables are free from multicollinearity, (iii): the residuals are normally distributed and (iv): the residuals do not display spatial autocorrelation [31,49,50].

Based on the results of the exploratory regression, we determined overall model significance, the presence of heteroscedasticity and a wide range of diagnostics by creating an OLS regression model in OpenGeoDa 1.2.0 [40] with the same dependent and explanatory variables as suggested by the exploratory regression.

#### Geographically Weighted Regression

Since the OLS regression is a global regression model, it estimates the strength of the relationship between the dependent variable and the explanatory variables averaged over the whole study area. However, the larger the study area, the more unlikely it is that one single coefficient per explanatory variable reflects the true underlying spatial relationship between the dependent variable and the explanatory variable since spatial data tend to vary over space. Global statistics tend to lead to the conclusion that relationships between variables are equal across the entire study area whereas local statistics can show the falsity of this assumption by displaying how the relationships vary across space [51]. The geographically weighted regression (GWR) method is therefore an extension to the traditional standard regression methodology and estimates a wide range of local parameters and diagnostics.

The Poisson distribution within the GWR framework is currently the most suitable for disease data, especially if observed counts of cases are low in specific areas [52–54]. The dependent variable was specified within the geographically weighted Poisson regression (GWPR) as the observed number of HCV cases per postal code and the offset variable was specified as the number of adult persons per postal code. The GWPR model calculates an additional global Poisson regression model, which can be compared to the results of the global OLS model to test the hypothesis that a Poisson regression is more suitable for HCV than the traditional OLS regression. The explanatory variables for the global and local Poisson regression models were the same variables that were found to be significant as specified by the OLS model. The centroids of each postal code were used as input coordinates. The GWPR model then uses a kernel and fits for each coordinate a regression equation where the coordinate in the centre of the kernel is the regression point. The data points inside the kernel are weighted from the centre of the kernel towards the edge of the kernel. Data points outside the kernel receive a weight of zero and are not included in the regression equation. For each coordinate, the data points are weighted differently so that each regression point has a unique regression equation. We used an adaptive kernel size so that in rural areas where data points are sparse, the kernel bandwidth will increase in size and will decrease in urban areas where data points are plentiful. The size of the bandwidth for each kernel and regression point is optimized using Akaike`s Information Criterion (AIC) [51]. To facilitate interpretation of the regression coefficients of the GWPR, the coefficients were exponentiated to show an increase or decrease of the relative risk of the dependent variable per one-unit change in the respective explanatory variable [52]. Statistical significance for each coefficient per postal code was calculated using pseudo t-values [51]. The statistic behind the GWPR method is described in detail elsewhere [52]. The computation of the GWPR was carried out using the GWR4 software [55].

## Results

### Spatial Distribution of Hepatitis C Prevalence among Adults

**Fig 1:**
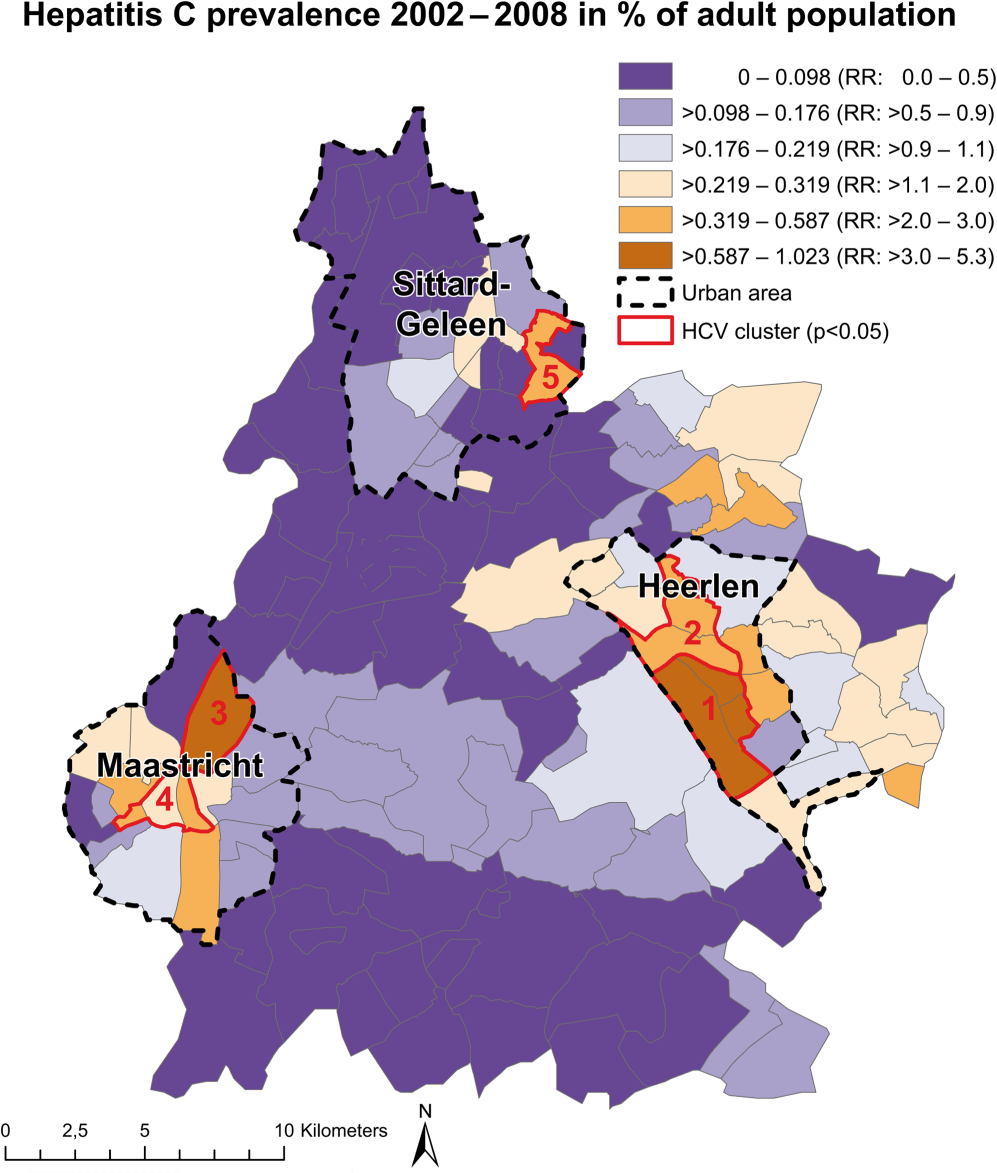
Spatial Distribution of HCV prevalence and RR, 2002–2008.

The prevalence and the risk estimates between the postal code areas varied widely, ranging from 0 to 1.02% of the adult population per postal code. The overall prevalence rate among adults was 0.19% of the total adult population. There was a clear urban-rural divide within the study area. Areas with higher risks were strongly concentrated within the urban areas of Heerlen, Maastricht and to a lower extent in Sittard-Geleen (Fig 1). Moran`s I revealed significant positive global autocorrelation of the HCV prevalence (Moran`s I = 0.43, p<0.001), indicating that postal codes with higher risks are close to each other.

The spatial scan statistic detected five significant local clusters (Fig 1). These are postal codes with statistically significant elevated risk of diagnosed HCV. All clusters could be observed within the three urban areas of the study area (Table 2). In total, these clusters contain 268 (34%) of all observed HCV infections in the study area.

**Table 2:** Significant clusters with high HCV risk as determined by the spatial scan statistic.

Cluster nr.	Location	RR	Cases	P-value
**1**	Southern part of Heerlen (3 postal codes)	4.30	91	<0.001
**2**	Northern part of Heerlen (2 postal codes)	2.83	60	<0.001
**3**	Northern part of Maastricht (1 postal code)	4.03	31	<0.001
**4**	Centre of Maastricht (3 postal codes)	1.91	71	<0.001
**5**	Eastern part of Sittard-Geleen (1 postal code)	3.29	15	<0.05

### Demographic and Socio-economic Determinants of HCV

As our analysis was exploratory in nature, we were interested in determining variable combinations based on population data that delivered a plausible explanation of HCV risk. We therefore identified two models that met all requirements for a properly specified OLS model that delivered a plausible explanation of the HCV prevalence. The AICc value of both OLS models differed only by 3, justifying a comparison of both models [56]. The first model consisted of the following explanatory variables that were overall positively associated with HCV risk: (i) proportion of divorced persons, (ii) proportion of one-person households, (iii) proportion of non-western immigrants and (iv) proportion of males aged 36–45. The second model consisted of the variables (i) average income per person, (ii) one-person households, (iii) mean property value and (iv) males aged 36–45. Variables for the second model were overall positively associated with HCV risk except for average income and mean property value, that both showed an inverse association. The same variables that were found to be significant in the OLS models were then used for further analysis in the global and local Poisson models.

By comparing model performance in terms of the goodness-of-fit AICc statistic (Table 3), the model with the lowest AICc value is the model with the best fit [24]. Based on this criterion, for both Model 1 and Model 2 the AICc value suggests that the global Poisson regression had a better fit than the OLS regression. However, the local Poisson regression outperformed both global regression approaches. The local Poisson regression of model 2 was the overall best-fitting regression model in terms of the AICc value as well as the percentage of local deviance explained.

**Table 3:** Comparison of global and local models.

Model	Local Deviance Explained	AICc
	**Model 1**	
**OLS**	0.50	495
**Global Poisson**	0.47	372
**Local Poisson**	0.53	334
	**Model 2**	
**OLS**	0.50	498
**Global Poisson**	0.48	360
**Local Poisson**	0.55	323

### Results of the Geographically Weighted Poisson Regression

#### Model 1

**Fig 2:**
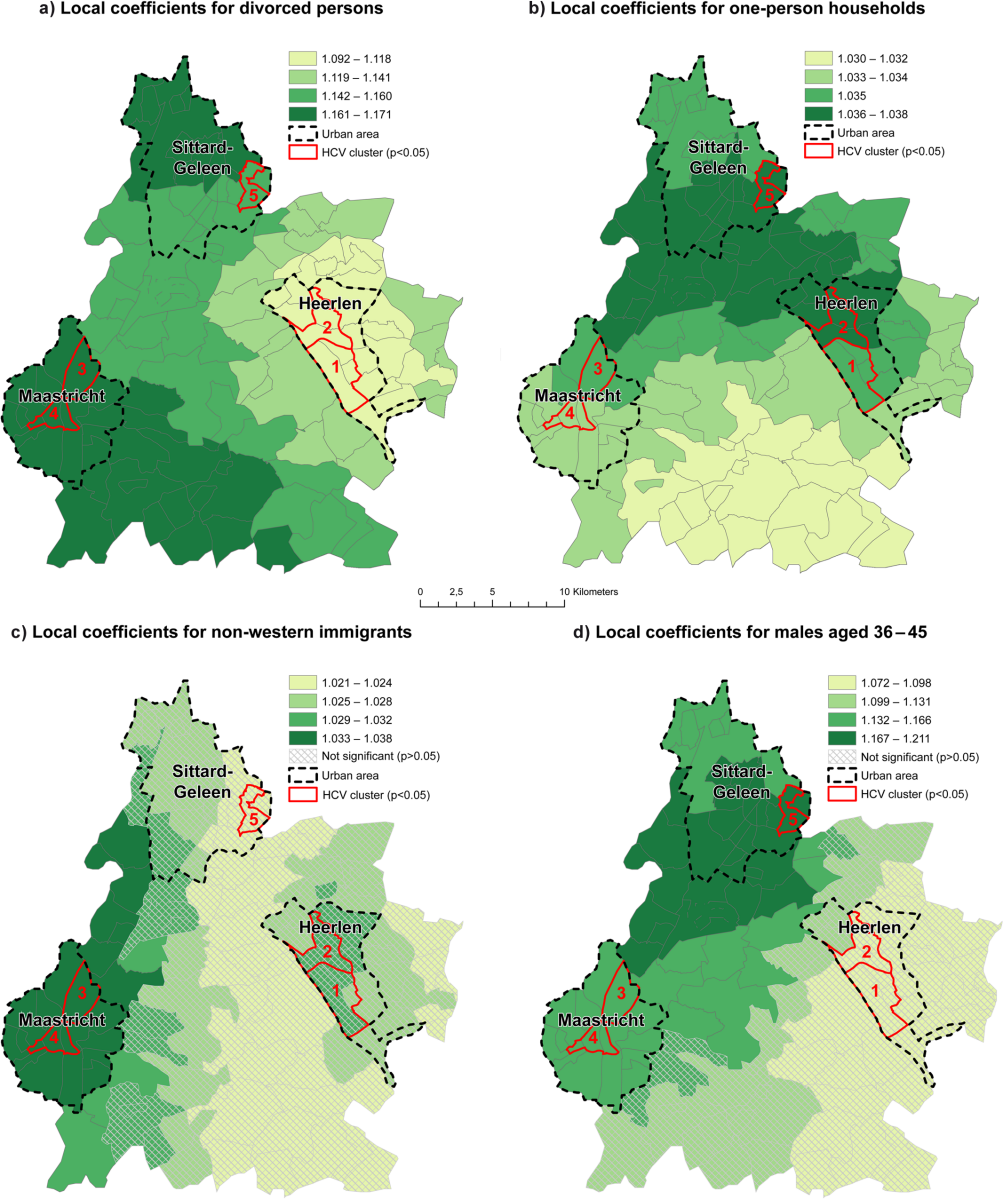
Local coefficients for model 1.

The results of the local Poisson model revealed strong local differences of the regression coefficients within the local clusters of elevated HCV risk (Table 4). The impact of the proportion of divorced persons on HCV risk was strongest in cluster 3 and 4 in Maastricht and cluster 5 in the northern part of Sittard-Geleen. In Heerlen, the impact of divorced persons was lowest (Fig 2). The impact of one-person households displayed intra-urban differences as well as regional differences. The association between the proportion of one-person households and HCV risk was strongest in the northern part of Heerlen (cluster 2) and the southern part of Sittard-Geleen (cluster 5). In cluster 3 and 4 in Maastricht, the impact of one-person households was overall lower than in the other urban areas and clusters. However, the northern part of Maastricht displayed a stronger association of one-person households to HCV risk than the southern part (Fig 2). The association between the proportion of non-western immigrants and HCV risk was only significant in cluster 3 and 4 in Maastricht and surrounding areas (Fig 2). Also, the association between the proportion of males aged 36–45 years and HCV risk displayed large regional differences; its impact was only significant in cluster 5 in Sittard-Geleen, followed by clusters 3 and 4 in Maastricht and the rural areas in between (Fig 2).

**Table 4:** Significant (p<0.05) coefficients per HCV cluster for model 1.

HCV Cluster nr.	Determinants	Coefficient
**1**	Divorced persons	1.102
	One-person households	1.034
**2**	Divorced persons	1.096
	One-person households	1.036
**3**	Divorced persons	1.161
	One-person households	1.035
	Non-western immigrants	1.036
	Males aged 36–45	1.157
**4**	Divorced persons	1.164
	One-person households	1.034
	Non-western immigrants	1.035
	Males aged 36–45	1.146
**5**	Divorced persons	1.159
	One-person households	1.036
	Males aged 36–45	1.161

#### Model 2

**Fig 3:**
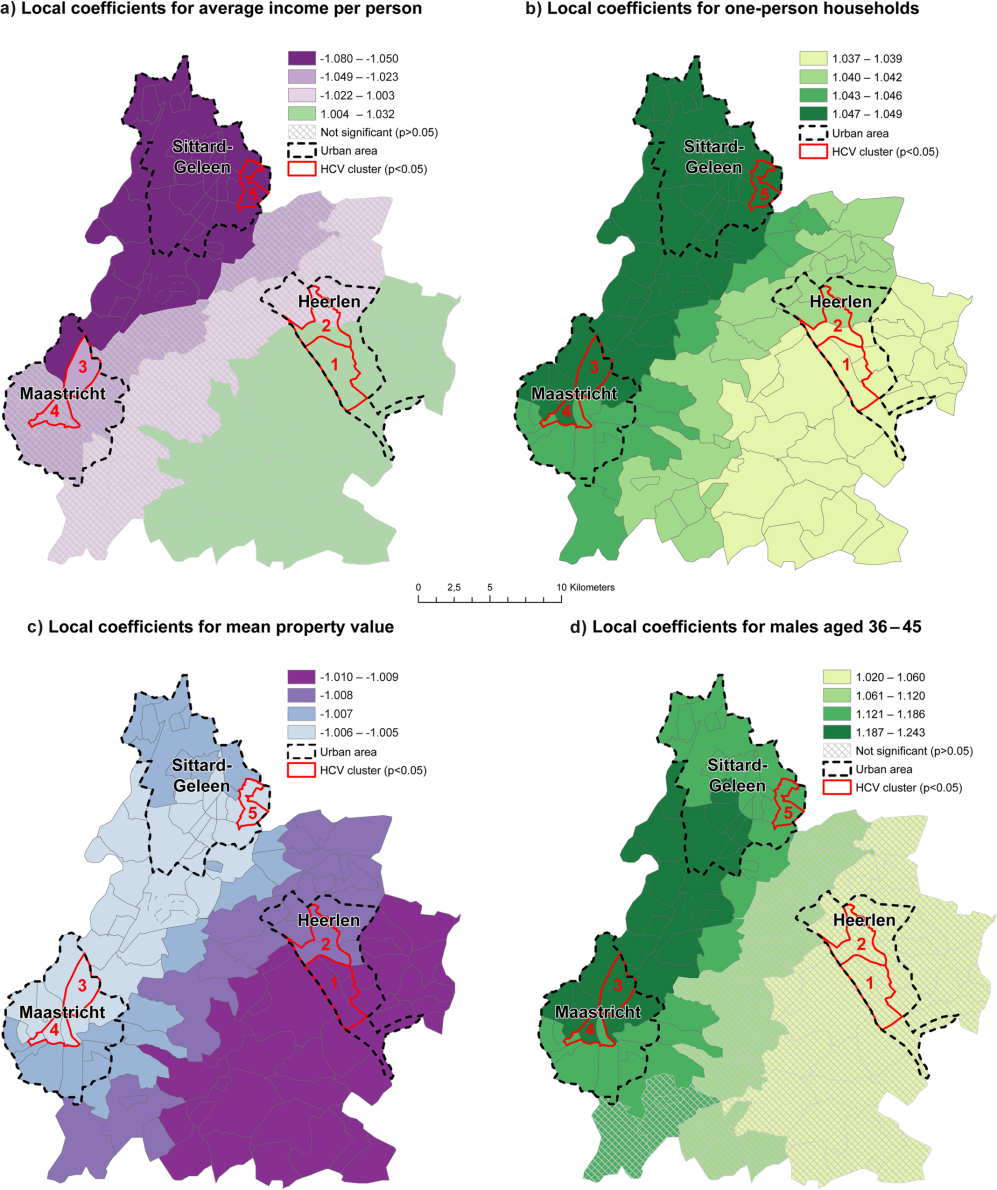
Local coefficients for model 2.

Comparable to the first model, the second model revealed strong local differences of the coefficients within the HCV clusters (Table 5). The association of HCV risk to average income was overall negative, indicating that a lower income is associated with a higher HCV risk. The local coefficients however, revealed that this association is not in the whole study area significant and negative. Average income is only significant inversely associated with HCV risk in cluster 5 in Sittard-Geleen and one postal code area in Maastricht (Fig 3). The proportion of one-person households was positively associated with HCV risk in cluster 5 in Sittard-Geleen and the northern postal codes of Maastricht in cluster 3. This association decreased in strength towards cluster 1 and 2 in Heerlen (Fig 3). Mean property value was negatively associated to HCV risk in all areas but the association displayed strong regional and intra-urban differences and was strongest in the southern postal codes of Heerlen in cluster 1 (Fig 3). The association between the proportion of males aged 36–45 and HCV risk displayed a similar pattern as observed in model 1. The association was only significant in the northern parts of Maastricht in cluster 3 and 4, the southwestern parts of Sittard-Geleen in cluster 5 and areas in between (Fig 3).

**Table 5:** Significant (p<0.05) coefficients per HCV cluster for model 2.

HCV Cluster nr.	Determinants	Coefficient
**1**	One-person households	1.038
	Mean property value	-1.009
**2**	One-person households	1.039
	Mean property value	-1.008
**3**	One-person households	1.047
	Mean property value	-1.006
	Males aged 36–45	1.213
**4**	One-person households	1.045
	Mean property value	-1.006
	Males aged 36–45	1.186
**5**	Average income per person	-1.070
	One-person households	1.046
	Mean property value	-1.006
	Males aged 36–45	1.161

## Discussion

The prevalence of HCV varies geographically within the province of South Limburg and clusters were located in urban areas. The main population at risk were divorced persons, male residents aged 36–45 and non-western immigrants residing in the area. Socio-economic determinants associated with HCV risk included one-person households, low income at individual level and areas with low mean property value. The associations between these determinants and HCV risk displayed strong regional and intra-urban differences.

The overall prevalence of diagnosed HCV cases was 0.19%, which is in the range of previous overall estimations of the HCV prevalence within the Dutch population [7,57]. However, the prevalence showed strong local variations with prevalences ranging between 0 and 1.023%,

Five local clusters of significantly elevated HCV risk were detected. These clusters were located in the three urban areas in the region. These results suggest that HCV risk is higher in urban areas than in rural areas and clusters geographically. Thereby, HCV prevalence does not only vary between countries, as was noted before [13,14] but also on small geographic scales such as postal code areas. The small-scale variation of HCV prevalence corresponds with findings of another spatial analysis of HCV in a higher prevalence country [58]. Local clustering of HCV prevalence in urban areas is typical for a wide range of infectious diseases, including HIV [26], *Neisseria gonorrhoea* [42] and *Chlamydia trachomatis* [27]. The detection of local clusters in our study may serve as a basis for prioritization of areas for future targeted and evidence-based screening interventions [26,42]. However, it should be noted that only a third of all HCV cases were detected in these clusters. The other cases showed a more random distribution over the region.

To what extent would these demographic and socio-economic determinants be of additional value to focus prevention strategies? When assuming that the population-based determinants represent the actual individual-based risk factors, then all determinants revealed here may indicate who are the key populations for HCV. Targeting these risk factors in the areas identified as clusters could serve as a practically applicable basis for prioritization of future screening interventions.

While there is a wide range of literature available about the prevalence of HCV infections and its associated risk factors [13,14], only a local analysis as employed here may help to understand the patterns of HCV infections and its associations to socio-economic determinants to effectively use available financial resources for targeted screening efforts.

The proportion of residents that were divorced was found to be associated with HCV risk over the complete study region. Marital status had been previously associated with HCV risk, yet findings were inconsistent [59–62]. Being divorced could be a proxy for sexual and economic instability. The found association between HCV risk and divorced persons may therefore serve as basis for future research on the role of marital status and potential high-risk sexual behaviour on HCV transmission in the study area. Non-western immigrants were identified as ethnic risk group in our study. Although this association corresponds well to previous studies focusing on risk factors of HCV in the Netherlands [15,16], the association of non-western immigrants to HCV risk was only significant in Maastricht. Potentially, in the other cities, immigrants from eastern-European countries might be more relevant as ethnic risk group [13,15].

Males aged 36–45 were another main demographic risk group identified in our analysis confirming US findings [3]. It is considered unlikely that this association can be for a large part explained by HIV positive MSM, as they comprise an important but only small part of the HCV cases in the Netherlands. [63]. However, the association between males aged 36–45 and HCV risk was only significant in the western part of the study area. One-person households were identified as a risk factor relating to household size. Although this association to HCV may not be obvious at first, it is in line with our findings that divorced persons are an overall risk factor for HCV and could be a potential additional proxy for sexual and economic instability. This finding may additionally serve as a basis for future research on the role of one-persons households and HCV transmission. Mean property value and low income at personal level were important socio-economic determinants associated with HCV risk [35] and are in line with other studies showing that low socio-economic status is an important risk factor for HCV [10,13,36]. However, our study demonstrated that low income at personal level was only significant in the urban area of Sittard-Geleen, while mean property value was found to be overall significant within the study area. Although this corresponds well to previous findings [10,13,36], it highlights the importance of including several markers for low socio-economic status on personal, household and area level to understand how these different measures of low socio-economic status impact the prevalence of HCV infections.

Several determinants were associated with HCV risk in the complete study region while others were only associated in certain regions; but all associations showed regional variance. The strong spatial differences observed suggest that the importance of demographic and socio-economic determinants to characterize the HCV key population may depend largely on the area where the HCV infected individual lives. Our findings are therefore in line with other studies applying GWR for infectious diseases [24,30,64].

In all clusters, an association was observed between HCV risk and divorced persons, one-person households and low mean property value. The proportion of middle-aged males were only associated to HCV in the clusters 3–5, and the proportion of non-western immigrants were only associated in the clusters 3 and 4. Income at personal level was only inversely associated in cluster 5. Thus, the impact of demographic and socio-economic determinants differed across the study area for the identified clusters.

## Limitations

First, the spatial analysis of this study was based on the four-digits postal code areas of the Netherlands. Although this spatial aggregation may be considered as a fine geographic scale [34], the prevalence rate of HCV follows the potentially arbitrary administrative boundaries of these postal codes. The results of our analysis might differ if a different level of aggregation had been chosen. This problem is often referred to as the modifiable areal unit problem (MAUP) and has not only an impact on the spatial distribution of HCV risk and the location of the detected clusters, but also on the results of the ecological regression analysis [65]. For our study, it would have been favourable to use street-level addresses of the HCV positive persons and underlying population at risk to analyse the spatial distribution of HCV without the limitation of arbitrary administrative boundaries [26]. This would not only allow a precise localization of HCV clusters, but could offer the chance to perform a geographically weighted logistic regression to provide more detailed insights on the spatially varying association between HCV risk and associated socio-economic and demographic determinants [51]. However, the HCV laboratory data as well as the population data used in this study were not available on this scale.

Second, it is unknown whether testing was motivated by the individuals due to symptoms related to HCV infection or was advised by a general practitioner due to prior knowledge of potential exposure factors of the tested individual. It is also unknown whether geographical, demographic or socio-economic determinants may have been associated with access to testing services (e.g. by distance, lack of knowledge, illiteracy) hence may have influenced the observed associations. The tested persons might therefore differ from the general population. During the initial data analysis, we tested the association of tested persons to demographic or socio-economic population characteristics through an additional exploratory regression model with the log-transformed percentage of tested persons as dependent variable. However, the exploratory regression analysis could not find demographic or socio-economic population characteristics that delivered a properly specified OLS regression model.

Additionally, we compared the spatial pattern of the ratio of HCV positive persons to tested persons with the ratio of positive persons to the adult population. Both approaches displayed a similar spatial pattern. An additional cluster analysis using a Bernoulli model in SaTScan with the number of negative tested persons as controls [45] could be used to test whether the location of spatial clusters will change when using the negative tested persons as denominator. This might additionally indicate, whether testing is performed randomly or follows different spatial patterns that cannot yet be explained by population or demographic characteristics that were available for this study. However, we applied only a Poisson model as our goal was to compare the HCV prevalence within our study area to previous estimates of the HCV prevalence in the Netherlands, which would not be possible when applying a case-control study design.

In our study, we consider the geographical spread of diagnosed HCV as a realistic representation of the diagnosed HCV prevalence among the adult population since the proportion of tested persons could not be properly explained by demographic or socio-economic population characteristics and the two compared ratios displayed a similar spatial pattern.

Third, the demographic and socio-economic determinants examined are practically applicable but are hampered by lack of precision as they are based on population data and not on an individual level. Population data provide population characteristics per neighbourhood. Therefore, additional research is needed to study whether the population-based determinants for key populations actually capture the individuals comprising such key population.

Fourth, we previously estimated that up to 66% of all HCV-positive patients in the study region were hidden to current screening practices [10]. As a result, cases that were diagnosed may differ from the cases that were still hidden with respect to the variables studied here.

Given the limitations outlined above, it is unknown to what extent the clusters and the demographic and socio-economic determinants really reflect the hidden population. A proof-of-principle intervention targeting postal codes in a detected cluster is currently being set up to reveal whether the hidden HCV infected individuals are appropriately addressed by our detected clusters and determinants. Additionally, we may have missed associations of potential determinants not captured in our analyses, as these were unavailable in the population databases such as educational level.

Also, the population-based determinants used in this study were taken from the Statline database 2009 as this was the earliest population data to include socio-economic variables and the customized stratified demographic data on sex and age were only available for 2012 but not for the years between 2002–2008. Although this might influence the results, it is unlikely that this has a strong impact as the demographic composition in the Netherlands remained relatively stable within the last few years [66].

The application of a Geographically Weighted Poisson regression clearly demonstrated spatial variability of the coefficients and underlined that future screening interventions for HCV clearly have to take into account the spatially varying association between demographic and socio-economic determinants. However, Paéz et al. point out that the use of GWPR delivers more robust results when applied on large datasets containing more than 160 administrative units [67]. Therefore, future research applying GWPR for HCV should focus on larger areas such as whole countries to gain more robust insights on the spatial variation of determinants for HCV [23,29,30]. The reproducibility of our study would allow a similar analysis for the whole of the Netherlands.

## Conclusions

In this study, we used spatial epidemiological methods to analyse the spatial distribution of HCV and its associated demographic and socio-economic determinants. Our results revealed strong regional differences not only of the HCV prevalence but also of the association between demographic and socio-economic determinants and HCV risk. These findings underline that a one-size-fits-all approach is not appropriate and that future screening interventions need to take into account the spatially varying demographic and socio-economic determinants for HCV. Our approach may not only be useful for South-Limburg, but may be useful in other countries as well.
